# Sequential Associations of Sleep Architecture, Nocturnal Oxygenation, and Respiratory Disturbance With a Composite PSG‐Derived Rhythm‐Event Index

**DOI:** 10.1002/brb3.71882

**Published:** 2026-10-28

**Authors:** Nasim Nikoubakht, Fatemeh kashaninasab, Mir Farhad Ghalehbandi, Mahboobeh Khoozan, Mohammad Ali Nazari

**Affiliations:** ^1^ Department of Anesthesiology, School of Medicine, Hazrat‐e Rasool General Hospital Iran University of Medical Sciences Tehran Iran; ^2^ Department of Psychiatry, Rasool Akram Medical Complex Clinical Research Development Center (RCRDC) Iran University of Medical Sciences Tehran Iran; ^3^ Department of Psychiatry, School of Medicine Mental Health Research Center, Psychosocial Health Research Institute Iran University of Medical Sciences Tehran Iran; ^4^ Mental Health Research Center, Department of Psychiatry, School of Medicine Iran University of Medical Sciences Tehran Iran; ^5^ School of Medicine Iran University of Medical Sciences Tehran Iran

## Abstract

**Background:**

Sleep‐disordered breathing (SDB), particularly obstructive sleep apnea (OSA), has been associated with cardiovascular complications including cardiac arrhythmias. However, the relative contributions of respiratory, oxygenation, and sleep architecture indices to composite PSG‐derived rhythm‐event index remain incompletely understood.

**Objective:**

To investigate the sequential associations of sleep architecture, nocturnal oxygenation, and respiratory disturbance indices with the composite PSG‐derived rhythm‐event index in patients undergoing polysomnography (PSG).

**Methods:**

Demographic, respiratory, oxygenation and sleep‐architecture variables were extracted from PSG records. The primary outcome was a composite PSG‐derived rhythm‐event count, estimated as round(arrhythmia index × TST_hours) from the PSG‐exported arrhythmia index, which was calculated during total sleep time only. Negative binomial regression used an offset of log(TST_hours). Because the outcome demonstrated overdispersion, negative binomial regression with an offset of log(TST hours) was used. Because the original 14‐predictor model was over‐parameterized for the sample size, a six‐predictor model (age, sex, BMI, AHI, T90, and sleep efficiency) was chosen post hoc. This reduced model was not prespecified before the original analysis. Sleep efficiency was retained because it showed a strong signal in the exploratory 14‐predictor model. The resulting *p*‐values should therefore be interpreted as optimistic and exploratory. A sequential model‐building approach (Model 1: age, sex, BMI, AHI; Model 2: plus T90; Model 3: plus sleep efficiency) was used to document the incremental contribution of each domain. A fully adjusted 14‐predictor model was fitted only as a supplementary analysis. Wald 95% CIs and bootstrap percentile 95% CIs (2000 resamples) are reported.

**Results:**

Forty‐one participants were included (mean age 47.8 ± 13.5 years; median BMI 29.4 kg/m^2^). Severe OSA (AHI ≥ 30 events/hour) was present in 56.1%. Median arrhythmia index was 3.2 events/hour, with marked overdispersion. In the six‐predictor model, only higher sleep efficiency was associated with lower arrhythmia rate (IRR = 0.946 per 1 percentage‐point increase, 95% CI 0.921–0.972, *p* < 0.001; bootstrap 95% CI 0.888–0.984). T90 was associated in the Wald inference (IRR = 1.034, 95% CI 1.006–1.063, *p* = 0.016) but this association was not robust under bootstrap resampling (95% CI 0.979–1.067). AHI was not associated with arrhythmia rate (IRR = 1.000, 95% CI 0.987–1.014, *p* = 0.954). Age, sex and BMI were also not significant. A 14‐predictor exploratory model had a marginally lower AIC (411.4) but a substantially higher BIC (438.8), consistent with overfitting.

**Conclusion:**

In this exploratory selected sleep‐center cohort, AHI and other respiratory‐event indices were not independently associated with the composite PSG‐derived rhythm‐event index. Only higher sleep efficiency showed a robust association with lower arrhythmia rate, but this finding is hypothesis‐generating and may reflect unmeasured confounding. These results require confirmation in larger prospective studies and should not guide clinical risk stratification.

## Graphical abstract

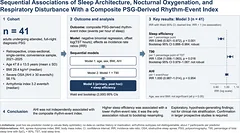

## 1 Introduction

Sleep‐disordered breathing (SDB), particularly obstructive sleep apnea (OSA), is one of the most common sleep‐related disorders and is characterized by recurrent episodes of upper airway obstruction during sleep associated with intermittent hypoxemia and sleep fragmentation (Peker et al. 2023; Redline et al. 2023).

OSA has become an important public health problem because of its high prevalence (ranged from 9% to 38%) and its strong association with cardiovascular morbidity and mortality (Gao et al. 2023; Senaratna et al. 2017).

Nocturnal hypoxemia and recurrent arousal episodes in OSA may lead to sympathetic nervous system activation, endothelial dysfunction, oxidative stress, hemodynamic instability, and systemic inflammation (Tetaj et al. 2026). These pathophysiological alterations may contribute to structural remodeling of the myocardium and increase susceptibility to cardiovascular complications, including cardiac arrhythmias (Maiolino et al. 2023; Tetaj et al. 2026).

Several polysomnographic and oxygenation indices, including the apnea–hypopnea index (AHI), oxygen desaturation index (ODI), and duration of oxygen saturation below 90%, are commonly used to evaluate the severity of some complications sleep‐disordered breathing and nocturnal hypoxemia (Maiolino et al. 2023).

Cardiac arrhythmias are increasingly recognized as important cardiovascular complications associated with OSA. Clinical and epidemiological studies consistently demonstrate a higher prevalence of atrial fibrillation and other rhythm disturbances in patients with OSA compared with individuals without sleep‐disordered breathing (Moula et al. 2022). In particular, OSA has been identified as an independent risk factor for the development and recurrence of atrial fibrillation in some cases, and its severity correlates with increased arrhythmia burden (Cadby et al. 2015; Moula et al. 2022; Wong et al. 2015).

Despite substantial evidence linking obstructive sleep apnea and cardiac arrhythmias, important gaps remain. Most previous studies have focused mainly on atrial fibrillation rather than overall arrhythmia burden, and have typically relied on single sleep metrics such as the apnea–hypopnea index (Dediu et al. 2015; Heinzinger et al. 2025; Heinzinger et al. 2023). In addition, the combined and independent effects of multiple sleep and oxygenation indices on the number of cardiac arrhythmias have not been well established. Furthermore, few studies have used statistical approaches that account for the interrelated nature of sleep data and patient‐level variability.

The aim of this study is to investigate the sequential and incremental associations of sleep architecture parameters, oxygenation indices, and respiratory disturbance measures with the number of cardiac arrhythmias in patients undergoing polysomnography. Rather than developing a definitive predictive model for clinical use, we sought to use a simple hierarchical modeling framework to identify which sleep‐ and oxygenation‐related variables provide independent explanatory value beyond demographic characteristics and standard indices of sleep‐disordered breathing (e.g., AHI). Because the number of candidate polysomnographic predictors exceeds what a single‐center cohort of this size can reliably support, the present study is explicitly exploratory. We chose a six‐predictor primary model post hoc (the a priori power calculation assumed five) and report the fully adjusted model only as a supplementary analysis to illustrate the instability introduced by over‐parameterization.

## 2 Methods

### 2.1 Study Design and Setting

This was a retrospective, cross‐sectional, descriptive‐analytical study conducted at the Sleep Center of Rasoul Akram Hospital, Iran University of Medical Sciences. The study examined the association between sleep architecture, nocturnal oxygenation indices, and sleep‐disordered breathing severity with a composite PSG‐derived rhythm‐event index in patients undergoing overnight PSG.

### 2.2 Participants

We retrospectively reviewed a convenience sample of eligible adult patients who underwent attended, full‐night diagnostic polysomnography at the Sleep Center of Rasoul Akram Hospital, Iran University of Medical Sciences, between 2021 and 2025. Patients were not randomly selected. Records were reviewed against prespecified inclusion and exclusion criteria, and only records meeting all eligibility criteria were retained for analysis. Because the sleep laboratory database did not contain a complete screening log, the exact number of records screened and the number excluded at each step could not be reconstructed. The final analytic sample comprised 41 participants.

The sleep laboratory database did not contain structured information on past medical history, pre‐existing arrhythmia, cardiovascular disease, major comorbidities, medication use (including beta‐blockers, antiarrhythmics, or other rate/rhythm‐controlling drugs), oxygen therapy, or the reason for PSG referral. These variables were therefore not available for summary or for sensitivity analysis.

Inclusion criteria were: (1) completion of a full‐night diagnostic polysomnography with technically adequate electroencephalographic, respiratory, oximetry, and electrocardiographic signals and (2) availability of complete clinical and polysomnographic data required for analysis. Exclusion criteria were: (1) markedly poor signal quality or incomplete recordings precluding reliable scoring of respiratory events or arrhythmias; (2) CPAP/BiPAP titration studies; (3) split‐night evaluations; and (4) missing key polysomnographic or clinical variables. The final analytic sample comprised 41 participants.

### 2.3 Polysomnography

All patients underwent standard overnight attended PSG using a multichannel system. Recorded signals included electroencephalography, electrooculography, electrocardiography, airflow (nasal pressure and/or thermistor), thoracoabdominal respiratory effort, pulse oximetry, and body position.

The following sleep architecture variables were derived:

- Total sleep time (TST), in minutes
- Sleep efficiency%: (TST/ total bed time) × 100
- Percentages of N1, N2, N3, and rapid eye movement (REM) sleep

### 2.4 Respiratory and Oxygenation Measures

The following indices were calculated:

- Apnea–hypopnea index (AHI): number of apneas plus hypopneas per hour of sleep
- Oxygen desaturation index (ODI): number of ≥3% oxygen desaturations per hour of sleep
- T90: percentage of total sleep time spent with peripheral oxygen saturation (SpO_2_) < 90%. For all regression models, T90 was analyzed as a continuous percentage variable. T90 in minutes was not used.

These variables were used as markers of sleep‐disordered breathing severity and nocturnal hypoxemia burden.

### 2.5 Arrhythmia Assessment

Cardiac rhythm was continuously monitored via a single‐lead electrocardiogram integrated into the polysomnography montage. All recordings were visually reviewed by an experienced sleep physician. The polysomnography system does not perform automated arrhythmia classification, and no per‐event or subtype labels (e.g., atrial fibrillation, premature atrial or ventricular complexes, bradyarrhythmias, or pauses) were recorded during scoring. The exported dataset therefore contained only an aggregate arrhythmia index, expressed as the number of arrhythmic events per hour of total sleep time; an event‐by‐event list was not available. The arrhythmia index was calculated during total sleep time only, not over total time in bed or the whole recording; events during wake were therefore not counted, and total sleep time was the denominator. No per‐event artifact annotations were available for systematic exclusion; the only safeguard against artifact was physician visual review. The outcome should therefore be regarded as a composite PSG‐derived rhythm‐event index rather than a validated per‐event arrhythmia count.

For descriptive and bivariate analyses, the arrhythmia index (events/hour) was used directly as a continuous measure. Because negative binomial regression requires a non‐negative integer count, an estimated total arrhythmia count was derived as round(arrhythmia index × TST_hours) and modeled with an offset of log(TST_hours). Because the index was calculated during total sleep time only, this product estimates the number of events during sleep. Coefficients are therefore interpretable as incidence rate ratios (IRRs) for the arrhythmia rate (events/hour). This count is a derived quantity and not a directly enumerated event list; arrhythmia subtypes could not be distinguished.

### 2.6 Sample Size

An original power calculation was performed before the study using G*Power based on a multiple linear regression with five predictors, an effect size of *f* ^2^ = 0.467, α = 0.05, and power = 0.95; this indicated a minimum of 34 participants. The final analytic sample comprised 41 participants because this was the number of eligible records with complete polysomnographic data after exclusions. Because the study was retrospective, attrition was not the reason for the final sample size. The post hoc six‐predictor model was not the basis of this calculation, and the study should be considered exploratory.

Because the power analysis was based on linear regression models whereas the final analyses used negative binomial regression for count outcomes, the calculated power may not perfectly reflect the primary modeling framework.

### 2.7 Statistical Analysis

Continuous variables are presented as mean ± standard deviation or median (interquartile range), as appropriate, and categorical variables as frequencies and percentages. Group comparisons, where applicable, were performed using parametric or non‐parametric tests according to data distribution.

The primary outcome was the nocturnal composite rhythm‐event count, derived by multiplying the polysomnography‐exported arrhythmia index (events/hour) by total sleep time in hours (index × TST_hours). Because this outcome was a discrete, overdispersed count variable (Pearson's *χ* ^2^/df ≈ 1.9–2.1), negative binomial regression with an offset of log(TST_hours) was used, and coefficients are interpreted as incidence rate ratios (IRRs) for arrhythmia rate (events/hour). The negative binomial dispersion parameter for the primary model was *θ* = 0.576 (SE = 0.114), corresponding to *α* = 1/*θ* = 1.737.

#### 2.7.1 Primary Model Specification

To improve the patient‐to‐predictor ratio (approximately 7:1), the six‐predictor primary analytic model was chosen post hoc. This model was not prespecified before the original analysis, and the a priori power calculation (G*Power; *f* ^2^ = 0.467, *α* = 0.05, power = 0.95, five predictors; minimum *n* = 34) did not refer to it. Sleep efficiency was selected as the sleep‐architecture predictor because it showed strong signal in the exploratory 14‐predictor model; *p*‐values and intervals for the primary model should therefore be regarded as optimistic. The primary model (Model 3) included age, sex, BMI, AHI, T90, and sleep efficiency. The outcome was the derived nocturnal arrhythmia count, *Y_i_* = round(arrhythmia index*_i_* × TST_hours_i_), modeled using negative binomial regression to account for overdispersion. Total sleep time (TST) was not entered as a predictor; it was included only as an offset/exposure denominator. The exact primary model was:

$$Y_{i}∼\mathrm{NB}\left(\mu_{i},\vartheta \right)$$

$$\begin{array}{ccc} \log\left(\mu_{i}\right) & = & \log\left(\mathrm{TS}T_{i}\right)+\beta_{0}+\beta_{1}\mathrm{Ag}e_{i}+\beta_{2}\mathrm{Femal}e_{i}+\beta_{3}\mathrm{BM}I_{i}\\ & & +\;\beta_{4}\mathrm{AH}I_{i}+\beta_{5}T90_{i}+\beta_{6}\mathrm{SleepEfficienc}y_{i} \end{array}$$

where TST*_i_* is total sleep time in hours, *θ* is the negative binomial dispersion parameter, and sex is coded with male as the reference category. Because TST is an offset, exp(*β_k_*) represents the incidence rate ratio (IRR) for arrhythmia rate (events/hour) per unit increase in predictor *k*.

A sequential model‐building approach was used to document the incremental contribution of each physiological domain:

1. Model 1 (demographic and respiratory model): age, sex, BMI, AHI (4 predictors).
2. Model 2 (oxygenation model): Model 1 plus T90 (5 predictors).
3. Model 3 (primary model): Model 2 plus sleep efficiency (6 predictors).

A fully adjusted exploratory model containing 14 predictors (Model 3 plus N1, N3/deep sleep, ODI, REM, PLM index, REM‐ and non‐REM‐specific EEG/EMG arousal indices, and supine time) was also fitted. Because this model exceeds the recommended patient‐to‐predictor ratio for count regression in a sample of this size, it is reported only as a supplementary analysis and is not used to draw inferential conclusions. To reduce collinearity among sleep‐stage percentages, N2 was omitted as the reference stage, and arousal indices were modeled using the REM‐ and non‐REM‐specific measures (A_EEG_EMG_REM, A_EEG_EMG_nonREM).

In sensitivity analyses, we also fitted three alternative oxygenation specifications: Model 2a (Model 1 + ODI), Model 2b (Model 1 + T90; identical to the primary Model 2), and Model 2c (Model 1 + ODI + T90). These are reported in Table S8. Because AHI and ODI were strongly collinear, Model 2c did not yield stable estimates and is presented for transparency only; it is not used for inferential conclusions.

For clinical interpretability, continuous predictors in the primary model were also expressed per clinically meaningful increments: sleep efficiency and T90 per 10 percentage‐point increase, and AHI per 10 events/hour. Because the negative binomial model uses a log link, these IRRs were obtained by exponentiating the per‐1‐unit coefficient multiplied by 10; confidence intervals were transformed similarly. Bootstrap percentile intervals (2000 resamples) were used as a robustness check.

#### 2.7.2 Selection Between ODI and T90

Because ODI and T90 both index nocturnal hypoxemia, we assessed whether they could be entered simultaneously in Model 2. They were moderately correlated (Spearman's ρ = 0.65, *p* < 0.001), but a negative binomial model containing both produced unstable estimates, and neither variable added explanatory value over the other by likelihood‐ratio test (adding T90 to the ODI model: *p* = 0.40; adding ODI to the T90 model: *p* = 0.27). AIC was marginally lower for ODI alone than for T90 alone (418.7 vs. 419.2; Table S8), a negligible difference, so T90 was chosen on the grounds given below. ODI was in addition strongly correlated with AHI (ρ = 0.87), indicating substantial redundancy with the primary severity index, whereas T90 carried information more distinct from AHI (ρ = 0.68). We therefore retained only T90 as the oxygenation predictor in Model 2 and present the ODI‐based specification as a sensitivity analysis in Table S8.

For transparency, the exploratory model deliberately retained both ODI and T90 despite their collinearity, so that the instability introduced by over‐parameterization is fully visible; this model is not used for inferential conclusions.

#### 2.7.3 Model Comparison and Inference

Model fit was compared using the Akaike information criterion (AIC) and Bayesian information criterion (BIC), where lower values indicate better fit, together with likelihood‐ratio tests for nested models. Wald 95% confidence intervals (CIs) are reported for all coefficients. Because profile‐likelihood intervals were unstable in this small sample, bootstrap percentile intervals (2000 resamples) were computed as a robustness check for the primary model. Wald standard errors condition on the estimated dispersion parameter and may be anticonservative in small samples. A two‐sided *p* < 0.05 was considered statistically significant. Collinearity was assessed using variance inflation factors (VIF); influential observations were examined using Cook's distance, and a sensitivity analysis excluding observations with Cook's *D* > 0.5 is reported in Table S7.

#### 2.7.4 Bivariate Analyses

Bivariate associations between the arrhythmia index and each predictor were assessed using Spearman's rank correlation for continuous variables and the Mann–Whitney *U* test for two‐group comparisons. To account for multiple comparisons, the false discovery rate (FDR) method of Benjamini and Hochberg was applied within each set of tests.

Collinearity was assessed using variance inflation factors (VIF). Influential observations were examined using Cook's distance. A sensitivity analysis excluding observations with Cook's *D* > 0.5 is reported in Table S7.

All analyses were performed using R software (version 4.4.2; R Foundation for Statistical Computing, Vienna, Austria).

### 2.8 Ethics

The study protocol was reviewed and approved by the Ethics Committee of Iran University of Medical Sciences (ethics ID: IR.IUMS.FMD.REC.1404.488). The study was conducted in accordance with the principles of the Declaration of Helsinki. To protect patient confidentiality, all extracted data were recorded in statistical files without names and only with unique identification codes. Access to the raw data was restricted to the principal investigator. No additional costs or non‐standard therapeutic interventions were imposed on patients, as the study was based solely on analysis of existing medical and polysomnographic records.

## 3 Results

### 3.1 Participant Characteristics

A total of 41 patients who underwent overnight polysomnography were included in the analysis (Figure 1). The median age was 48.0 years (interquartile range [IQR] 15.0; mean 47.8 ± 13.5 years), and the median BMI was 29.4 kg/m^2^ (IQR 5.6), indicating that most participants were overweight or obese (Table 1).

**FIGURE 1:**
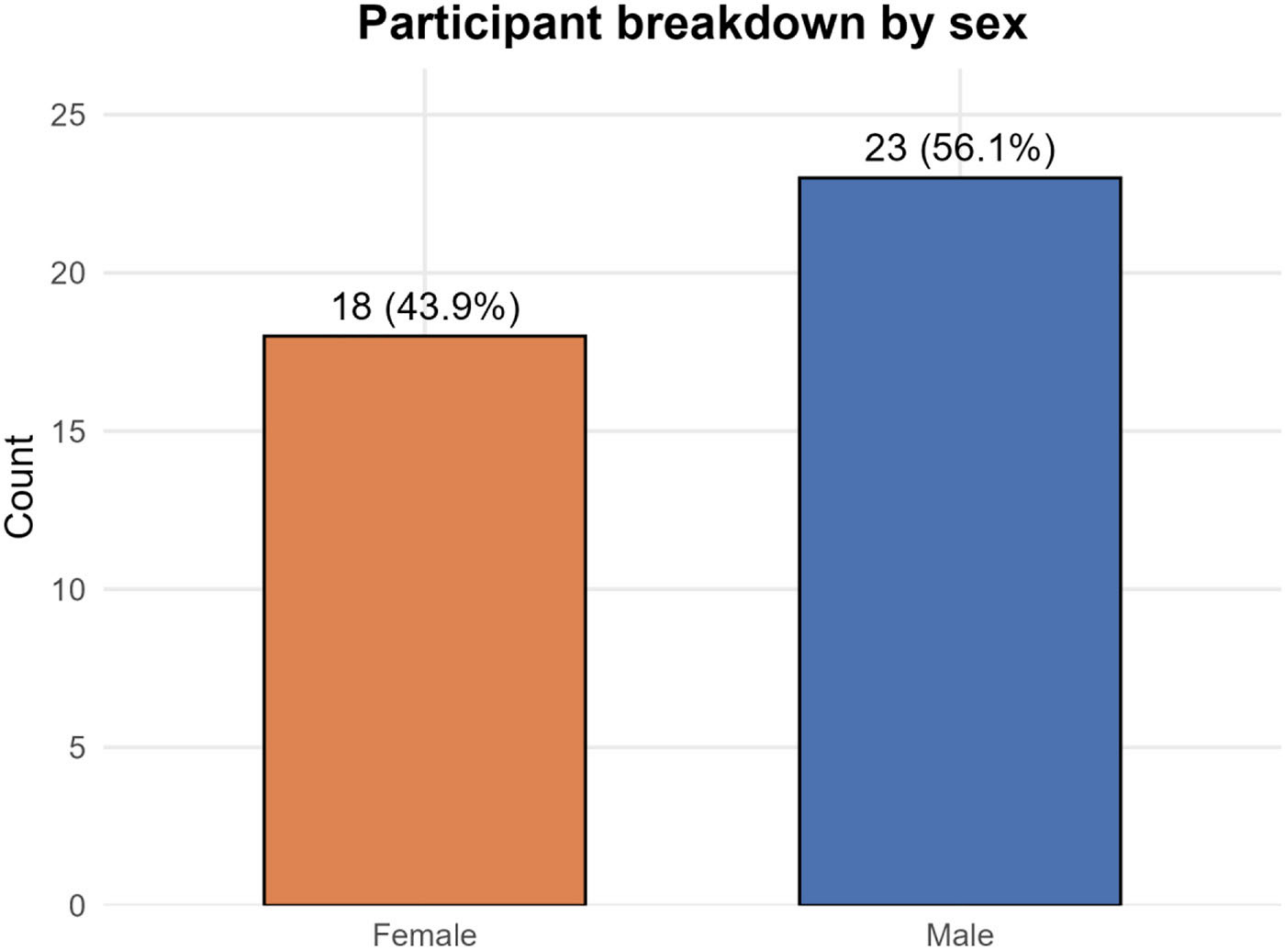
Participant breakdown by sex.

**TABLE 1:** Descriptive characteristics of the study population (*n* = 41).

Variable	*N*	Median	IQR	Mean	SD
Age (years)	41	48.0	15.0	47.83	13.54
BMI	41	29.4	5.6	30.47	5.40
AHI	41	36.0	48.4	45.57	38.96
T90 (% of TST)	41	2.7	14.3	12.78	19.01
ODI	41	22.9	34.2	29.46	30.29
Arrhythmia	41	3.2	10.5	13.86	27.27
Sleep_Efficiency	41	88.6	12.0	83.30	16.80
Deep_Sleep	41	22.2	14.8	23.00	13.66
PLM	41	5.9	16.8	11.98	15.51
Total_Sleep_Time	41	328.0	44.3	312.67	72.44
N1	41	12.1	9.8	16.01	15.72
N2	41	52.5	17.9	52.33	15.97
REM	41	8.1	13.1	8.67	8.28
A_EEG_EMG_REM	41	60.0	85.3	51.50	41.89
A_EEG_EMG_nonREM	41	65.2	41.3	60.19	29.98
Supine	41	47.1	36.2	48.47	25.85

*Notes*: Values are presented as median and interquartile range (IQR) and mean ± standard deviation (SD).

Abbreviations: BMI: body mass index (kg/m^2^); AHI: apnea–hypopnea index (events/hour); T90 (% of TST): percentage of total sleep time with oxygen saturation < 90%; ODI: oxygen desaturation index (events/hour); Arrhythmia: nocturnal cardiac arrhythmia index (events/hour); Sleep_Efficiency: ratio of total sleep time to time in bed (%); Deep_Sleep: total duration of N3 (slow‐wave) sleep (% of total sleep time); PLM: periodic limb movement index (events/hour); Total_Sleep_Time: total sleep time (minutes); N1, N2: non‐rapid eye movement (NREM) sleep stages 1 and 2 (% of total sleep time); REM: rapid eye movement sleep (% of total sleep time); A_EEG_EMG_REM: EEG/EMG arousal index during REM sleep (events/hour); A_EEG_EMG_nonREM: EEG/EMG arousal index during NREM sleep (events/hour); SUPINE: percentage of total sleep time spent in the supine position.

### 3.2 Sleep‐Disordered Breathing and Nocturnal Oxygenation

Obstructive sleep apnea severity was predominantly high. Based on the AHI, 2 patients (4.9%) had normal or near‐normal values (AHI < 5), 9 (22.0%) had mild, 7 (17.1%) had moderate, and 23 (56.1%) had severe OSA (AHI ≥ 30). AHI had a median of 36.0 events/hour (Figure 2).

**FIGURE 2:**
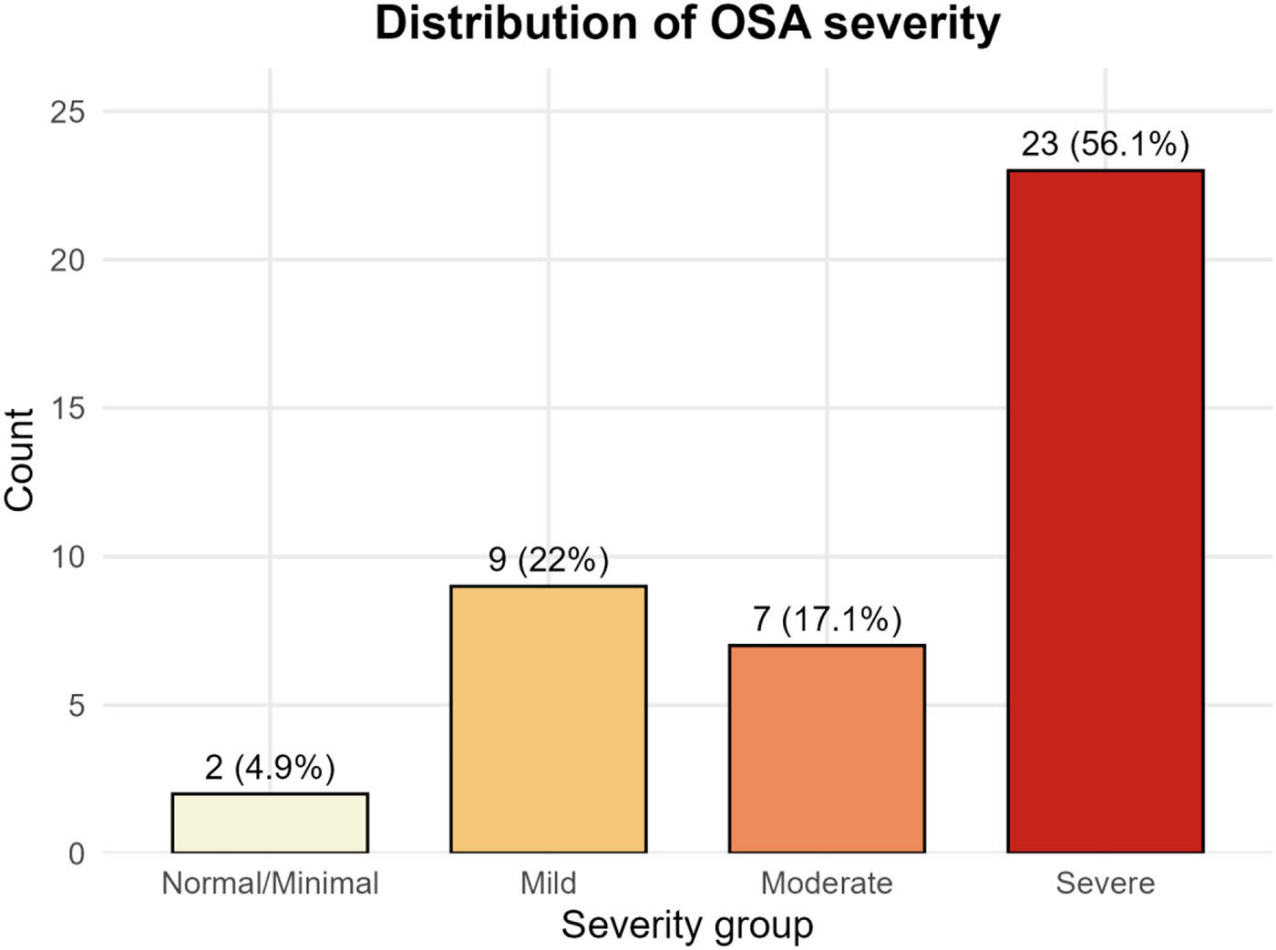
Distribution of OSA severity.

Nocturnal oxygenation indices showed a right‐skewed distribution. The median percentage of total sleep time spent with SpO_2_< 90% (T90) was 2.7%, whereas the mean T90 was 12.8%, indicating that a subset of patients experienced marked hypoxemia. The median ODI was 22.9 events/hour.

### 3.3 Sleep Architecture

Median TST was 328 min (approximately 5.5 h), with an IQR of 44.3 min. Median sleep efficiency was 88.6%, and the mean was 83.3%. The median proportion of N3 (slow‐wave) sleep was 22.2% of TST. The periodic limb movement (PLM) index had a median of 5.9 events/hour.

### 3.4 Arrhythmia Index (Event per Hour)

The PSG‐exported arrhythmia index (events/hour), calculated during total sleep time, was used for descriptive analyses. The primary outcome for the negative binomial model was an estimated composite rhythm‐event count, derived by multiplying this index by total sleep time in hours; the product was rounded, round(arrhythmia index × TST_hours), and modeled with an offset of log(TST_hours). The distribution of the arrhythmia index was highly skewed and overdispersed: the median arrhythmia index was 3.2 events/hour (IQR 10.5) and the mean index was 13.9 events/hour, with a variance‐to‐mean ratio of 53.7, indicating marked overdispersion. Thus, while 19 patients (46.3%) had fewer than 3 arrhythmias per hour, a small subset exhibited a markedly elevated arrhythmia index.

For descriptive purposes, the arrhythmia index (events/hour) is reported, as this unit is more clinically interpretable than the derived count. The negative binomial model used the derived count with an offset of log(TST hours), yielding IRRs that are also expressed per hour of sleep.

### 3.5 Bivariate Associations

Spearman's rank correlations (*n* = 41) revealed one significant unadjusted association with the arrhythmia index: an inverse correlation with total sleep time (ρ = −0.316, *p* = 0.044). No other sleep or respiratory variable reached statistical significance, although REM sleep showed a negative trend (ρ = −0.288, *p* = 0.068), and both N1 (ρ = 0.265, *p* = 0.094) and sleep efficiency (ρ = −0.265, *p* = 0.095) approached but did not attain conventional significance. After false discovery rate (FDR) correction for multiple testing, none of the correlations remained significant (all adjusted *p* > 0.05) (Table 2).

**TABLE 2:** Correlations (Spearman's ρ) between sleep, physiological, and demographic predictors and arrhythmia index (*n* = 41).

Predictor	ρ	*p*	*p*_ADJ_FDR
Total_Sleep_Time	−0.316	0.0440	0.378
REM	−0.288	0.0680	0.378
N1	0.265	0.0939	0.378
Sleep_Efficiency	−0.265	0.0946	0.378
PLM	0.208	0.1909	0.464
BMI	−0.192	0.2280	0.464
Supine	−0.191	0.2321	0.464
A_EEG_EMG_REM	−0.156	0.3296	0.586
ODI	−0.120	0.4546	0.682
N2	−0.116	0.4690	0.682
AGE	0.083	0.6056	0.771
A_EEG_EMG_nonREM	0.078	0.6266	0.771
AHI	−0.067	0.6753	0.772
T90 (% TST)	−0.051	0.7533	0.804
Deep_Sleep	−0.024	0.8839	0.884

Abbreviations: AHI, apnea–hypopnea index; BMI, body mass index; EEG, electroencephalography; EMG, electromyography; nonREM, non‐rapid eye movement; ODI, oxygen desaturation index; PLM, periodic limb movement; REM, rapid eye movement.

When the arrhythmia index was compared by sex, no difference was observed (Mann–Whitney *p* = 0.237).

### 3.6 Multiple Negative Binomial Regression

Model fit statistics are summarized in Tables 3 and S2. Model 1 (age, sex, BMI, AHI) had AIC = 417.4 and BIC = 427.7. Adding T90 (Model 2) did not improve fit (AIC = 419.2; BIC = 431.2; LRT *p* = 0.72). Adding sleep efficiency (Model 3) produced the best‐fitting parsimonious model (AIC = 412.1; BIC = 425.8; LRT vs. Model 2, *p* = 0.002). A 14‐predictor exploratory model had a marginally lower AIC (411.4) but a substantially higher BIC (438.8), indicating overfitting; it was therefore retained only as a supplementary analysis (Table S3). The likelihood‐ratio test of Model 3 against the 14‐predictor model gave *p* ≈ 0.03 (8 degrees of freedom), so the evidence of overfitting rests on the BIC and the patient‐to‐predictor ratio rather than on the likelihood‐ratio test.

**TABLE 3:** Negative binomial regression for nocturnal arrhythmia rate (events/hour): sequential models (*n* = 41).

Predictor	Model 1 IRR (95% CI)	*p*	Model 2 IRR (95% CI)	*p*	Model 3 (primary) IRR (95% CI)	*p*
Age (years)	1.024 (0.990–1.059)	0.174	1.022 (0.989–1.057)	0.198	1.024 (0.992–1.056)	0.142
Sex (female vs. male)	1.194 (0.471–3.026)	0.709	1.184 (0.468–2.998)	0.722	0.922 (0.392–2.172)	0.853
BMI (kg/m^2^)	0.996 (0.906–1.095)	0.935	0.983 (0.891–1.085)	0.734	0.917 (0.835–1.008)	0.072
AHI (events/hour)	1.002 (0.989–1.016)	0.721	1.001 (0.987–1.016)	0.884	1.000 (0.987–1.014)	0.954
T90 (% TST)	—	—	1.007 (0.978–1.036)	0.652	**1.034 (1.006–1.063)**	**0.016**
Sleep efficiency (%)	—	—	—	—	**0.946 (0.921–0.972)**	**<0.001**
AIC	417.37		419.24		412.06	
BIC	427.65		431.24		425.76	

*Note*: The outcome was the nocturnal arrhythmia rate (events/hour), operationalized as an estimated arrhythmia count (arrhythmia index × TST hours) modeled with an offset of log(TST hours); incidence rate ratios (IRRs) therefore express the multiplicative change in arrhythmia rate per one‐unit increase in the predictor (or between exposure categories for sex). Model 1 = age, sex, BMI, AHI; Model 2 = Model 1 + T90; Model 3 = Model 2 + sleep efficiency (primary model, chosen post hoc). Wald 95% confidence intervals (CIs) are shown.

Dispersion parameter *θ* = 0.576 (SE = 0.114); *α* = 1/*θ* = 1.737. Pearson's *χ* ^2^/df ≈ 1.9–2.1.

Abbreviations: AHI, apnea–hypopnea index; AIC, Akaike information criterion; BIC, Bayesian information criterion; BMI, body mass index; CI, confidence interval; IRR, incidence rate ratio; T90, percentage of total sleep time with oxygen saturation < 90%; TST, total sleep time.

To express continuous‐variable effects using clinically meaningful increments, the per‐1‐unit estimates from the primary model correspond to the following. A 10 percentage‐point higher sleep efficiency was associated with a lower arrhythmia rate (IRR = 0.57, 95% CI 0.44–0.75; bootstrap 95% CI 0.31–0.85). A 10 percentage‐point higher T90 was associated with a higher arrhythmia rate under Wald inference (IRR = 1.40, 95% CI 1.06–1.84), but this association was not robust under bootstrap resampling (95% CI 0.81–1.91). AHI was not associated with arrhythmia rate (IRR = 1.00 per 10 events/hour, 95% CI 0.88–1.15). These increments confirm that only sleep efficiency remained robustly associated with arrhythmia rate (Table S9).

Thus, the oxygenation block did not demonstrate incremental value beyond demographic and AHI variables, whereas adding sleep efficiency improved model fit; however, only sleep efficiency remained robust under bootstrap resampling.

In the primary Model 3 (Table 3), higher sleep efficiency was associated with a lower arrhythmia rate (IRR = 0.946 per 1 percentage‐point increase, 95% CI 0.921–0.972, *p* < 0.001), and this association was robust to bootstrap resampling (bootstrap 95% CI 0.888–0.984) (Table S4). T90 was associated with a higher arrhythmia rate in the Wald inference (IRR = 1.034, 95% CI 1.006–1.063, *p* = 0.016) (figure 3) but the bootstrap 95% CI included 1 (0.979–1.067), indicating that this association was not robust. AHI was not associated with arrhythmia rate (IRR = 1.000, 95% CI 0.987–1.014, *p* = 0.954). Age, sex and BMI were also not significant (all *p* > 0.05). VIF values in Model 3 were all below 2, indicating no meaningful collinearity (Table S5). Cook's distance identified two potentially influential observations (*D* > 0.5) (Table S6); exclusion sensitivity analysis is reported in Table S7. After excluding them, the sleep‐efficiency association remained significant (IRR 0.961, *p* = 0.001), the T90 association was attenuated (IRR 1.011, *p* = 0.405), and the BMI association became statistically significant (IRR 0.854, 95% CI 0.781–0.932, *p* < 0.001).

**FIGURE 3:**
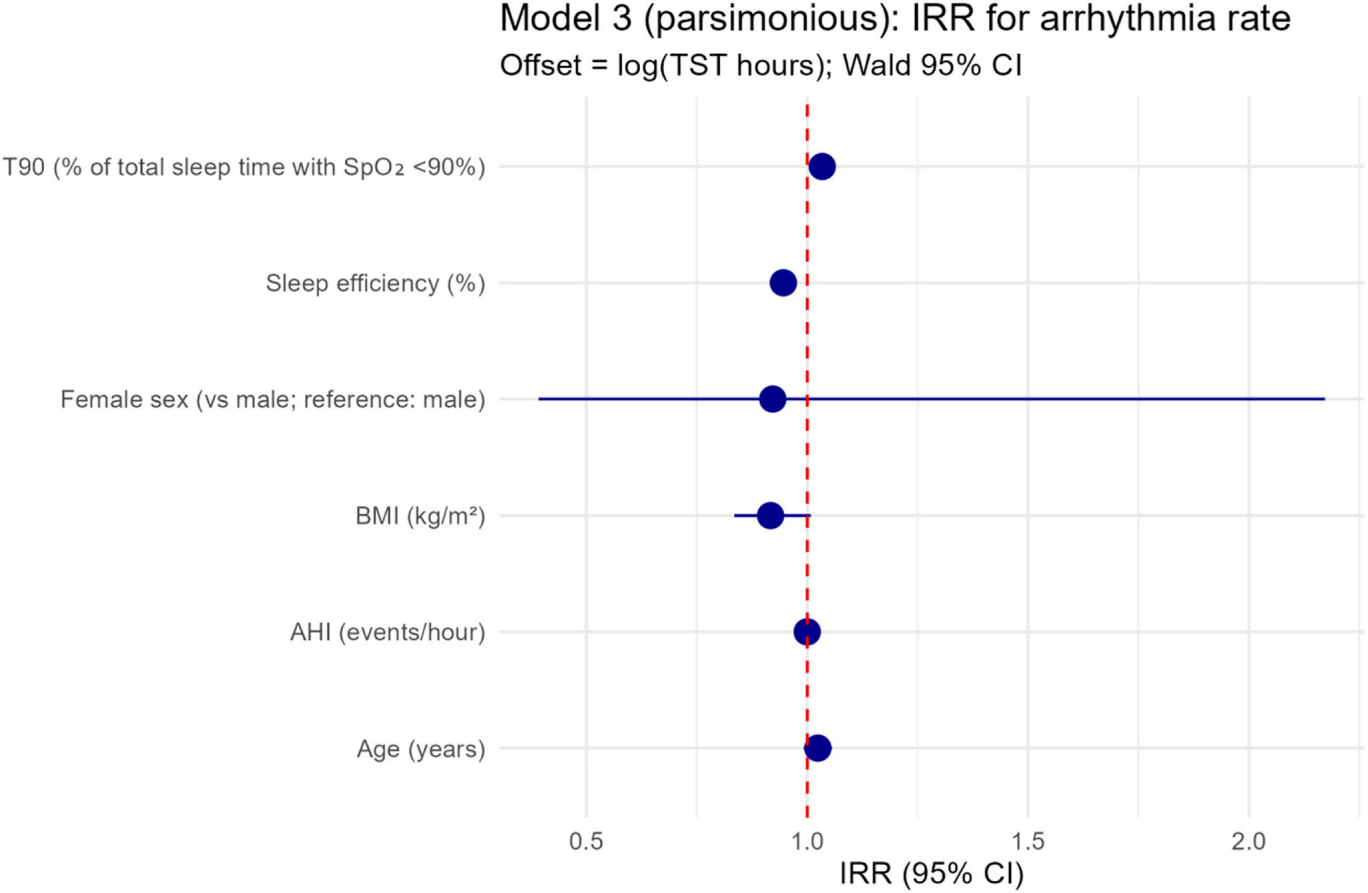
Incidence rate ratios from the primary parsimonious negative binomial model (Model 3). The model included age (years), sex (female vs. male; reference: male), BMI (kg/m^2^), AHI (events/hour), T90 (% of total sleep time with SpO_2_ <90%), and sleep efficiency (%). Total sleep time (hours) was included as an offset [log(TST hours)]. IRRs represent the multiplicative change in arrhythmia rate (events/hour) per one‐unit increase for continuous variables, or female versus male for sex. Wald 95% CIs are shown. T90 was analyzed as a percentage of total sleep time, not as minutes. Only sleep efficiency remained robustly associated with arrhythmia rate. Abbreviations: AHI, apnea–hypopnea index; BMI, body mass index; CI, confidence interval; IRR, incidence rate ratio; SpO_2_, peripheral oxygen saturation; T90, percentage of total sleep time with SpO_2_ <90%; TST, total sleep time.

## 4 Discussion

In this exploratory cohort, we found that AHI was not independently associated with the composite rhythm‐event index in any parsimonious specification. Among the polysomnographic variables examined, only higher sleep efficiency was robustly associated with lower arrhythmia rate, surviving both Wald and bootstrap inference. T90 was associated in the Wald model but not under bootstrap resampling, and the associations with N3 and other sleep‐architecture variables observed in a fully adjusted exploratory model did not survive model reduction. Together, these findings indicate that in a small single‐center cohort such as ours, most apparent associations between polysomnographic indices and the rhythm‐event index are not statistically stable.

Large cohorts have demonstrated that higher AHI is associated with incident AF and nocturnal arrhythmias in different populations, including patients with and without heart failure (Cadby et al. 2015; Guilleminault et al. 1983; Namtvedt et al. 2011; Olmetti et al. 2008; Roche et al. 2003). These observations underscore the established link between OSA severity, as indexed by AHI, and arrhythmia risk in larger cohorts, and provide important context for interpreting our findings.

The growing argument that AHI alone is an incomplete measure of clinically relevant OSA severity (Borsini et al. 2018; Malhotra et al. 2021) is not directly supported by our data. AHI was not associated with the rhythm‐event index in any specification, and T90 was associated only under Wald inference, not under bootstrap resampling. Our findings therefore do not provide robust evidence that hypoxaemic burden outweighs respiratory event frequency in this cohort. Studies such as Azarbarzin et al. (2019), Zeng et al. (2025), and Huang et al. (2023) have reported that hypoxic burden predicts cardiovascular outcomes in much larger populations; our null and unstable results are more plausibly attributable to limited statistical power than to a genuine absence of effect, but we cannot distinguish the two in this dataset.

Although T90 was associated under Wald inference, the oxygenation block did not improve model fit, and the association was not robust under bootstrap resampling. We therefore do not interpret T90 as providing incremental explanatory value in this dataset.

The literature supports a role for sleep architecture and sleep quality in cardiovascular risk. Zhang et al. (2022) and Heinzinger et al. (2025) reported associations between sleep‐architecture disruption, particularly reduced slow‐wave sleep, and cardiovascular risk or incident AF. In our cohort, N3 proportion was associated with  rhythm‐event count only in the over‐parameterized 14‐predictor exploratory model. This association did not persist after parsimonious modeling and we therefore do not interpret it as a substantive finding. It is more plausibly explained by overfitting or residual confounding in the small exploratory specification.

Higher sleep efficiency was associated with a lower rhythm‐event rate in our study, and this was the only association that remained robust under bootstrap resampling and after exclusion of influential observations. This is broadly consistent with a prior study linking poor sleep efficiency to AF and incident arrhythmias (Kwon et al. 2015), and suggests that sleep‐continuity measures may carry information relevant to arrhythmia burden in this population. In clinically meaningful terms, a 10 percentage‐point higher sleep efficiency corresponded to an approximately 43% lower arrhythmia rate in the primary model. This was the only association robust to bootstrap resampling and exclusion of influential observations. Because the arrhythmia index was calculated during total sleep time only, with total sleep time as the denominator, this association is not an arithmetic consequence of events during wake being divided by sleep time.

This study used a cross‐sectional, single‐center design in a small sample of patients undergoing polysomnography, which allows assessment of associations but precludes causal inference and limits generalizability. The modest sample size restricts statistical power, particularly for models with several correlated predictors, and increases the risk of model instability and overfitting. For this reason, we chose a six‐predictor primary model post hoc and treated the 14‐predictor model as exploratory only.

Negative binomial regression was methodologically appropriate for the overdispersed count outcome, and the sequential approach (demographic → oxygenation → sleep architecture) was useful for documenting the incremental contribution of each physiological domain. In the parsimonious primary model, VIF values were all below 2, indicating no meaningful collinearity. The fully adjusted 14‐predictor model nonetheless showed evidence of overfitting: although its AIC was marginally lower than the primary model (411.4 vs. 412.1), its BIC was substantially higher (438.8 vs. 425.8), and several coefficients had wide and unstable confidence intervals. Coefficients from that model should therefore not be interpreted inferentially.

A minimum sample size was calculated a priori using G*Power based on a multiple linear regression analysis of pilot data (*f* ^2^ = 0.467, power = 0.95, *α* = 0.05, five predictors; minimum *n* = 34; the final sample of 41 reflected the number of eligible records with complete data, not attrition). Although this formal calculation was performed, the power analysis used linear regression models, whereas the final analysis employed negative binomial regression for the count outcome. This methodological difference, combined with the pragmatic availability of patients, means the sample size may not be perfectly optimized for the primary analytic framework. Because the a priori calculation was based on five predictors, the six‐predictor primary model was chosen post hoc, and the larger exploratory model was reported only as a supplementary analysis.

Clinically, our findings do not support replacing AHI with oxygenation‐burden metrics for arrhythmia‐risk stratification on the basis of this dataset. The only association robust to bootstrap resampling was between higher sleep efficiency and lower arrhythmia rate, which suggests that sleep‐continuity measures may be worth carrying forward as candidate predictors in larger cohorts. Any clinical recommendation would require confirmation in prospective studies with adequate statistical power. In interpreting PLM results, note that the PLM index was calculated over total time in bed rather than total sleep time. This choice may dilute the PLM rate in participants with more wake after sleep onset and could partly explain the weaker association with arrhythmia in our models.

From a research perspective, these exploratory findings underscore the need for larger, longitudinal studies using appropriate count‐data models to determine whether sleep‐architecture measures, and in particular sleep continuity, can modify the nocturnal rhythm‐event rate and downstream cardiovascular outcomes. Future studies may also consider complementary metrics such as hypoxic burden (HB) (Azarbarzin et al. 2019; Huang et al. 2023; Zeng et al. 2025) and the sleep breathing impairment index (SBII) when examining associations between sleep‐disordered breathing and nocturnal arrhythmias.

This study has several strengths and limitations that merit consideration. Strengths include the use of full‐night, attended polysomnography with standardized scoring, and the modeling of the composite rhythm‐event count as an outcome using negative binomial regression, which appropriately accounted for overdispersion. However, the cross‐sectional, single‐center design precludes causal inference and limits generalizability beyond similar referral populations.

### 4.1 Arrhythmia Outcome

The arrhythmia outcome was derived from an index (events/hour) exported by the polysomnography software and converted to an estimated count by multiplying by total sleep time. Direct event‐by‐event enumeration was not possible, and the single‐lead ECG did not allow reliable arrhythmia subtyping. We were therefore unable to distinguish atrial fibrillation, premature atrial or ventricular complexes, bradyarrhythmias, or pauses, and could not report type‐specific associations. This is a substantial limitation: different arrhythmia subtypes have distinct mechanisms and prognostic implications, and aggregating them may attenuate or obscure subtype‐specific relationships with sleep and oxygenation metrics. Future studies using multi‐lead or Holter‐synchronized ECG with automated arrhythmia classification are needed.

### 4.2 Unmeasured Cardiac Confounders

The sleep laboratory database did not contain structured information on prior cardiac disease, prior atrial fibrillation, heart failure, echocardiographic parameters, or use of beta‐blockers, antiarrhythmic agents, or other rate/rhythm‐affecting medications. These are strong potential confounders of the relationship between sleep‐disordered breathing and arrhythmia burden. Their absence may bias the observed associations, in particular, the inverse associations with BMI and female sex could partly reflect confounding by heart failure, medication use, or referral patterns rather than a protective biological effect. All adjusted estimates should therefore be interpreted as exploratory and hypothesis‐generating, and the single robust association observed, between sleep efficiency and arrhythmia rate, may still be subject to residual confounding by unmeasured cardiac disease or medication use.

### 4.3 Sampling

Participants were a convenience sample of eligible records rather than a formally randomized or consecutively enrolled cohort; a complete screening log was not retained, which limits assessment of selection bias.

### 4.4 Patient‐to‐Predictor Ratio

Even the parsimonious six‐predictor model operates at ∼7 participants per predictor, which is at the lower limit of what negative binomial regression can reliably support. The 14‐predictor exploratory model operates at ∼3:1 and is therefore reported only for transparency.

### 4.5 Influential Observations

Cook's distance identified two observations with *D* > 0.5 in the primary model. Sensitivity analysis excluding these (*n* = 39) is reported in Table S7. The sleep‐efficiency association remained significant (IRR 0.946 → 0.961; *p* < 0.001 → *p* = 0.001), whereas the T90 association was attenuated and no longer significant (IRR 1.034 → 1.011; *p* = 0.016 → *p* = 0.405). The BMI association became statistically significant (IRR 0.854, *p* < 0.001) and the IRR for female sex fell to 0.506 (*p* = 0.070), indicating that estimates other than that for sleep efficiency are sensitive to these two observations.

### 4.6 Post Hoc Model Reduction

The six‐predictor model was derived after the original 14‐predictor analysis, and sleep efficiency was retained because it showed a strong signal in that model. Estimates, *p*‐values and bootstrap intervals for the primary model are therefore likely to be optimistic, and the finding requires confirmation in an independent sample.

### 4.7 Bootstrap Instability

Wide bootstrap intervals for T90, BMI, sex and age indicate that these coefficients are not reliably estimated in this sample. Conclusions should therefore rest only on the sleep‐efficiency finding.

### 4.8 ODI–T90 Collinearity

ODI and T90 could not be entered together, and ODI was strongly correlated with AHI. This limited our ability to distinguish frequency versus burden of hypoxaemia.

## 5 Conclusion

In this exploratory selected sleep‐center cohort (*n* = 41), AHI and other respiratory‐event indices were not independently associated with the composite PSG‐derived rhythm‐event index in the reduced parsimonious model. Only higher sleep efficiency was robustly associated with lower arrhythmia rate, but this finding is exploratory and may be influenced by unmeasured confounders such as pre‐existing cardiovascular disease, arrhythmia, and medication use. These results should not guide clinical risk stratification and require validation in larger prospective studies with complete confounder data.

## Author Contributions

**Nasim Nikoubakht**: conceptualization; methodology; supervision; writing, review and editing. **Fatemeh Kashaninasab**: investigation; data curation; writing, review and editing. **Mir Farhad Ghalehbandi**: methodology; formal analysis; review and editing. **Mahboobeh Khoozan**: validation; supervision. **Mohammad Ali Nazari**: investigation; data curation; formal analysis; visualization; writing the original draft. All authors: approved the final manuscript and agree to be accountable for all aspects of the work.

## Funding

The authors have nothing to report.

## Conflicts of Interest

The authors declare that they have no conflicts of interest.

## AI Disclosure

In accordance with journal guidelines, we disclose that artificial intelligence (large language models) was utilized solely for the purpose of English language editing, formatting, and proofreading to enhance the readability of this manuscript. The authors reviewed and edited all AI‐assisted text and take full responsibility for the scientific integrity, accuracy, and final content of the work.

## Supporting information




**Supplementary Material**: brb371882‐sup‐0001‐SuppMat.docx

## Data Availability

The data that support the findings of this study are available from the corresponding author upon reasonable request.
