# Supplementary material for: Sequential Associations of Sleep Architecture, Nocturnal Oxygenation, and Respiratory Disturbance With a Composite PSG‐Derived Rhythm‐Event Index
Source: Brain Behav. 2026 Oct 28;16(11):e71882. doi: 10.1002/brb3.71882 (PMC13717065; doi:10.1002/brb3.71882)
Supplement: Supplementary file 1 [file BRB3-16-e71882-s001.docx]

## Supplementary: Supplementary Table S1. Correlations among hypoxemia metrics (AHI, ODI, T90)

| Metric | AHI | ODI | T90 |
| --- | --- | --- | --- |
| AHI | 1.000 | 0.868 | 0.682 |
| ODI | 0.868 | 1.000 | 0.652 |
| T90 | 0.682 | 0.652 | 1.000 |

**Footnote:** Values are Spearman’s rho. All correlations were statistically significant at p < 0.001.
**Abbreviations:** AHI, apnea–hypopnea index; ODI, oxygen desaturation index; T90, percentage of total sleep time with oxygen saturation < 90%.

**Supplementary Table S2. Model fit statistics for hierarchical and exploratory models**

| Model | Predictors | AIC | BIC | df_resid | LRT vs previous model |
| --- | --- | --- | --- | --- | --- |
| Model 1 | Age, sex, BMI, AHI | 417.37 | 427.65 | 36 | Reference |
| Model 2 | Model 1 + T90 | 419.24 | 431.24 | 35 | p = 0.719 |
| Model 3 (primary) | Model 2 + sleep efficiency | 412.06 | 425.76 | 34 | p = 0.002 |
| Full exploratory | 14 predictors | 411.42 | 438.84 | 26 | p = 0.034 |

**Footnote:** Lower AIC/BIC indicates better fit. The full exploratory model had a marginally lower AIC but a substantially higher BIC, consistent with overfitting. LRT compares each model with the previous one; the full exploratory model is compared with Model 3.
**Abbreviations:** AIC, Akaike information criterion; BIC, Bayesian information criterion; LRT, likelihood-ratio test; BMI, body mass index; AHI, apnea–hypopnea index; T90, percentage of total sleep time with oxygen saturation < 90%.

**Supplementary Table S3. Full exploratory 14-predictor negative binomial regression model**

| Term | IRR | 95% CI | p |
| --- | --- | --- | --- |
| Age (years) | 1.027 | 0.996–1.059 | 0.089 |
| Sex (Female vs Male) | 0.336 | 0.129–0.873 | 0.025 |
| BMI (kg/m²) | 0.902 | 0.824–0.989 | 0.027 |
| AHI (events/hour) | 1.015 | 0.995–1.036 | 0.147 |
| T90 (% TST) | 1.031 | 1.005–1.058 | 0.019 |
| ODI (events/hour) | 1.006 | 0.980–1.032 | 0.673 |
| Sleep efficiency (%) | 0.892 | 0.839–0.947 | <0.001 |
| Deep sleep / N3 (% TST) | 1.063 | 1.026–1.101 | 0.001 |
| PLM index (events/hour) | 1.000 | 0.971–1.029 | 0.984 |
| N1 (% TST) | 0.972 | 0.912–1.036 | 0.386 |
| REM (% TST) | 1.046 | 0.987–1.109 | 0.128 |
| Arousal index, REM (events/hour) | 1.001 | 0.989–1.013 | 0.880 |
| Arousal index, non-REM (events/hour) | 1.015 | 0.994–1.036 | 0.171 |
| Supine time (% TST) | 1.020 | 1.002–1.038 | 0.034 |

**Model fit:** AIC = 411.42; BIC = 438.84.
**Footnote:** This model is over-parameterized for the available sample size and is reported only as a supplementary exploratory analysis. Coefficients should not be interpreted inferentially.
**Abbreviations:** IRR, incidence rate ratio; CI, confidence interval; BMI, body mass index; AHI, apnea–hypopnea index; T90, percentage of total sleep time with oxygen saturation < 90%; ODI, oxygen desaturation index; TST, total sleep time; PLM, periodic limb movement; REM, rapid eye movement; N1, non-REM stage 1; N3, non-REM stage 3.

**Supplementary Table S4. Bootstrap percentile 95% CIs for the primary Model 3**

| Term | Wald IRR | Wald 95% CI | Bootstrap IRR | Bootstrap 95% CI |
| --- | --- | --- | --- | --- |
| Age (years) | 1.024 | 0.992–1.056 | 1.026 | 0.991–1.065 |
| Sex (Female vs Male) | 0.922 | 0.392–2.172 | 0.798 | 0.222–2.590 |
| BMI (kg/m²) | 0.917 | 0.835–1.008 | 0.905 | 0.780–1.054 |
| AHI (events/hour) | 1.000 | 0.987–1.014 | 1.000 | 0.973–1.021 |
| T90 (% TST) | 1.034 | 1.006–1.063 | 1.026 | 0.979–1.067 |
| Sleep efficiency (%) | 0.946 | 0.921–0.972 | 0.949 | 0.888–0.984 |

**Footnote:** Bootstrap percentile intervals are based on 2,000 resamples. The T90 association was significant under Wald inference but not under bootstrap resampling.
**Abbreviations:** IRR, incidence rate ratio; CI, confidence interval; BMI, body mass index; AHI, apnea–hypopnea index; T90, percentage of total sleep time with oxygen saturation < 90%; TST, total sleep time.

**Supplementary Table S5. Collinearity diagnostics: VIF for Model 3 and Model 2c**

| Term | VIF (Model 3) | VIF (Model 2c) | Tolerance (Model 2c) |
| --- | --- | --- | --- |
| Age | 1.059 | 1.044 | 0.958 |
| Sex | 1.095 | 1.092 | 0.916 |
| BMI | 1.522 | 1.434 | 0.697 |
| AHI | 1.672 | 4.333 | 0.231 |
| T90 | 1.591 | 1.505 | 0.665 |
| Sleep efficiency | 1.190 | — | — |
| ODI | — | 4.318 | 0.232 |

**Footnote:** In Model 3, all VIF values were below 2, indicating no meaningful collinearity. In Model 2c, AHI and ODI showed VIF > 4, consistent with substantial collinearity when both respiratory and oxygenation metrics are entered together.
**Model 2c = Model 1 + ODI + T90**

**Abbreviations:** VIF, variance inflation factor; BMI, body mass index; AHI, apnea–hypopnea index; T90, percentage of total sleep time with oxygen saturation < 90%; ODI, oxygen desaturation index.

**Supplementary Table S6. Cook’s distance for the primary Model 3**

| Observation | Cook’s D |
| --- | --- |
| 1 | 0.0048 |
| 2 | 0.0144 |
| 3 | 0.0110 |
| 4 | 0.0010 |
| 5 | 0.1872 |
| 6 | 0.0006 |
| 7 | 0.0107 |
| 8 | 0.0034 |
| 9 | 0.0004 |
| 10 | 0.0222 |
| 11 | 0.0017 |
| 12 | 0.0108 |
| 13 | 0.0008 |
| 14 | 0.0033 |
| 15 | 0.0004 |
| 16 | 0.0076 |
| 17 | 0.0082 |
| 18 | 0.0022 |
| 19 | 0.0044 |
| 20 | 0.0072 |
| 21 | 0.0324 |
| 22 | 0.0103 |
| 23 | 0.0062 |
| 24 | 0.0027 |
| 25 | 0.0044 |
| 26 | 0.0499 |
| 27 | 0.0098 |
| 28 | 0.0610 |
| 29 | 0.0057 |
| 30 | 0.0164 |
| 31 | 0.0083 |
| 32 | 0.0104 |
| 33 | 0.5172 |
| 34 | 0.5159 |
| 35 | 0.0044 |
| 36 | 0.0000 |
| 37 | 0.0095 |
| 38 | 0.0074 |
| 39 | 0.1741 |
| 40 | 0.0190 |
| 41 | 0.0351 |

**Footnote:** Two observations (rows 33 and 34) had Cook's D > 0.5. Sensitivity analysis excluding these observations is reported in Supplementary Table S7. The sleep-efficiency association remained significant (IRR 0.946 → 0.961; p < 0.001 → p = 0.001), whereas the T90 association was attenuated and no longer significant (IRR 1.034 → 1.011; p = 0.016 → p = 0.405). BMI became statistically significant (IRR 0.854, 95% CI 0.781–0.932, p < 0.001), and the IRR for female sex fell to 0.506 (p = 0.070), indicating that estimates other than that for sleep efficiency are sensitive to these two observations.
**Abbreviation:** Cook’s D, Cook’s distance.

**Supplementary Table S7. Sensitivity analysis for the primary model (Model 3) excluding influential observations (Cook's D > 0.5)**

| Term | Full Model 3 (n = 41) |  |  |  | Excluding influential observations (n = 39) |  |  |  |
| --- | --- | --- | --- | --- | --- | --- | --- | --- |
|  | **IRR** | **95% CI** | **p** |  | **IRR** | **95% CI** | **p** |  |
| Age (per year) | 1.024 | 0.992–1.056 | 0.142 |  | 1.026 | 0.999–1.053 | 0.058 |  |
| Sex (Female vs Male) | 0.922 | 0.392–2.172 | 0.853 |  | 0.506 | 0.242–1.057 | 0.070 |  |
| BMI (per kg/m²) | 0.917 | 0.835–1.008 | 0.072 |  | 0.854 | 0.781–0.932 | <0.001 |  |
| AHI (per event/hour) | 1.000 | 0.987–1.014 | 0.954 |  | 1.011 | 1.000–1.023 | 0.058 |  |
| T90 (per % TST) | 1.034 | 1.006–1.063 | 0.016 |  | 1.011 | 0.986–1.036 | 0.405 |  |
| Sleep efficiency (per %) | 0.946 | 0.921–0.972 | <0.001 |  | 0.961 | 0.940–0.983 | 0.001 |  |
| Model fit |  |  |  |  |  |  |  |  |
| AIC | 412.06 |  |  |  | 363.18 |  |  |  |
| BIC | 425.76 |  |  |  | 376.49 |  |  |  |

**Footnote:** The outcome was the estimated nocturnal arrhythmia count (arrhythmia index × TST hours), modelled with negative binomial regression and an offset of log(TST hours). In the sensitivity analysis, two observations with Cook’s D > 0.5 were excluded (full model n = 41; sensitivity model n = 39). The sleep-efficiency association remained significant (IRR 0.946 → 0.961; p < 0.001 → p = 0.001). The T90 association was attenuated and no longer significant (IRR 1.034 → 1.011; p = 0.016 → p = 0.405). In contrast, BMI became statistically significant (IRR 0.854, 95% CI 0.781–0.932, p < 0.001), and the IRR for female sex fell to 0.506 (p = 0.070). These changes indicate that estimates other than the sleep-efficiency association are sensitive to these two influential observations and should be interpreted with caution given the small sample. Wald 95% CIs are shown.

Note: n = 39; not comparable with n = 41

**Abbreviations:** AHI, apnea–hypopnea index; AIC, Akaike information criterion; BIC, Bayesian information criterion; BMI, body mass index; CI, confidence interval; Cook’s D, Cook’s distance; IRR, incidence rate ratio; T90, percentage of total sleep time with oxygen saturation < 90%; TST, total sleep time.

**Supplementary Table S8. Alternative oxygenation specifications (Models 2a–2c)**

| Model | Oxygenation predictor(s) | IRR (95% CI) | p | AIC | BIC |
| --- | --- | --- | --- | --- | --- |
| Model 2a | ODI only | ODI: 0.973 (0.944–1.003) | 0.074 | 418.72 | 430.72 |
| Model 2b | T90 only | T90: 1.007 (0.978–1.036) | 0.652 | 419.24 | 431.24 |
| Model 2c | ODI + T90 | ODI: 0.960 (0.932–0.990); T90: 1.016 (0.988–1.046) | ODI: 0.009; T90: 0.263 | 420.02 | 433.73 |

**Footnote:** Model 2a = Model 1 + ODI; Model 2b = Model 1 + T90 and is identical to the primary Model 2 in the main text; Model 2c = Model 1 + ODI + T90. Model 2c includes both ODI and T90; because AHI and ODI were strongly collinear (Spearman ρ = 0.87; VIF > 4 for both), estimates from Model 2c are unstable and are presented for transparency only. They should not be interpreted inferentially. AIC and BIC are shown for descriptive purposes; lower values indicate better fit.

**Abbreviations:** AHI, apnea–hypopnea index; AIC, Akaike information criterion; BIC, Bayesian information criterion; CI, confidence interval; IRR, incidence rate ratio; ODI, oxygen desaturation index; T90, percentage of total sleep time with oxygen saturation <90%; VIF, variance inflation factor.

**Figure S1. Incidence rate ratios from the 14-predictor exploratory negative binomial model.**

Forest plot of incidence rate ratios (IRRs) and Wald 95% confidence intervals for each predictor in the fully adjusted 14-predictor model. The model is over-parameterised relative to the available sample size (approximately three participants per predictor) and is reported for transparency only. The width of the confidence intervals reflects this low patient-to-predictor ratio. Coefficients from this model should not be interpreted inferentially, and no clinical or causal inference is drawn from this figure.

**Figure S2. Spearman correlations among AHI, ODI, and T90.**

Heatmap of Spearman rank correlations among the three respiratory and oxygenation indices. Cell values are Spearman's rho. Correlations are shown for descriptive purposes only. No adjustment for multiple comparisons was applied within this figure, and the correlations should not be interpreted as evidence of a causal or directional relationship.

**Supplementary Table S9. Clinically meaningful increments for continuous predictors in the primary model (Model 3).**

| Predictor | Increment | IRR (Wald 95% CI) | Bootstrap 95% CI | p |
| --- | --- | --- | --- | --- |
| Sleep efficiency | per 10 percentage points | 0.57 (0.44–0.75) | 0.31–0.85 | <0.001 |
| T90 | per 10 percentage points of TST | 1.40 (1.06–1.84) | 0.81–1.91 | 0.016 |
| AHI | per 10 events/hour | 1.00 (0.88–1.15) | — | 0.954 |

Footnote: Derived from the primary Model 3 per-1-unit estimates. Because the model uses a log link, per-10-unit IRRs were obtained by exponentiating the per-1-unit coefficient multiplied by 10. T90 = percentage of total sleep time with SpO₂ < 90%; AHI = apnea–hypopnea index; TST = total sleep time.
